# Safety of Platelet-rich Plasma Treatment for Oral Diseases

**DOI:** 10.14789/ejmj.JMJ24-0054-OA

**Published:** 2025-06-06

**Authors:** MORIKUNI TOBITA, ANNA ARITA, YOSUKE MASUBUCHI, KEIKO WAKANA, HIKARI YONEDA, SHUNSUKE NAMAKI, TAKAAKI TAMAGAWA, MITSUYO SHINOHARA

**Affiliations:** 1 Medical Technology Innovation Center, Juntendo University, Tokyo, Japan; 1 Medical Technology Innovation Center, Juntendo University, Tokyo, Japan; 2 Clinical Trial and Research Center, Juntendo University Hospital, Tokyo, Japan; 2 Clinical Trial and Research Center, Juntendo University Hospital, Tokyo, Japan; 3 Department of Oral and Maxillofacial Surgery, Juntendo University Faculty of Medicine, Tokyo, Japan; 3 Department of Oral and Maxillofacial Surgery, Juntendo University Faculty of Medicine, Tokyo, Japan; 4 Department of Oral and Maxillofacial Surgery Ⅱ, Nihon University School of Dentistry, Tokyo, Japan; 4 Department of Oral and Maxillofacial Surgery Ⅱ, Nihon University School of Dentistry, Tokyo, Japan

**Keywords:** platelet-rich plasma, regenerative medicine, oral disease, safety

## Abstract

**Objectives:**

Platelet-rich plasma (PRP), obtained by centrifuging autologous blood, is widely used for pain relief and wound healing. PRP is also used in the dental field, but the safety and validity thereof for dental diseases have not yet been fully established. We aimed to evaluate the safety of PRP for the following dental procedures: post-extraction socket healing, maxillary sinus floor elevation, periodontal tissue regeneration, and intentional tooth replantation.

**Materials:**

PRP was purified from patient’s own venous blood and transplanted.

**Methods:**

Eight patients were enrolled in clinical trials targeting four dental diseases, and subjected to the required treatment with transplantation (two patients/study). The primary endpoint was safety; specifically, adverse events after PRP treatment were evaluated based on clinical observations. Secondary endpoints were clinical evaluation, dental radiography and/or imaging evaluation, and platelet count.

**Results:**

No serious adverse events associated with the PRP transplantation were observed. In tooth extraction sockets, postoperative pain tended to subside the day after treatment. Regeneration of hard tissue at the PRP transplant site has been confirmed in clinical studies on maxillary sinus lifts, periodontal tissue regeneration, and tooth replantation.

**Conclusions:**

Our findings demonstrate the safety and validity of PRP transplantation for each type of dental disease. Because the sample size was limited, further large-scale clinical studies are required to evaluate the safety and validity of PRP treatment for each dental disease.

## Introduction

Platelet-rich plasma (PRP) therapy is a regenerative medicine technology used worldwide for pain relief and tissue regeneration in conditions such as knee osteoarthritis^1)^, intractable ulcers^2)^, and alopecia^3)^. PRP is a plasma component containing numerous platelets and is manufactured by the centrifugation of anticoagulant added autologous peripheral blood. Although the mechanism of action of PRP has not been fully elucidated, it is thought that following activation of the platelets, multiple cytokines and growth factors are released from the α-granules into the affected tissues at supraphysiological concentrations, contributing to tissue repair and anti-inflammatory effects.

PRP is widely used in the dental field^4)^ to regenerate tissue lost due to periodontal disease and to promote wound healing and regeneration of hard tissue after tooth extraction and dental implant treatment^5)^. Although recent reports have shown that PRP transplantation may reduce postoperative pain and discomfort after tooth extraction and prevent the development of osteitis^6)^, the clinical significance and validity of PRP therapy for each dental disease have not been fully verified in clinical trials. PRP therapy is considered a relatively safe treatment because it uses autologous blood and the procedure is simple; however, adverse events (AEs) suspected to be related to PRP administration have been reported^7)^. Hence, further verification of the safety of PRP treatment for dental diseases is required. We, therefore, aimed to perform an exploratory evaluation of the safety and validity of PRP using the following dental procedures: 1) post-extraction sockets (socket preservation); 2) periodontal regenerative therapy; 3) maxillary sinus lift; and 4) tooth replantation.

## Materials and Methods

PRP transplantation was performed for four dental diseases. These were single-arm, open-label studies. Prior to beginning the study, approval was obtained from the Tokyo Medical and Dental University Specially Certified Committee for Regenerative Medicine (committee number: NA8140003, approval number: RM2018-008, RM2018-09, RM2018-010, RM2018-012) and the studies were registered in the Japan Registry of Clinical Trials (https://jrct.niph.go.jp/, registry number: jRCTc030190273, jRCTc030190274, jRCTc030190275, jRCTc030190277). Informed consent was obtained from all patients. Details of each protocol have been described previously (Tables 1 and 2)^8)^. This study was conducted between March 2019 and October 2023 in the Department of Oral and Maxillofacial Surgery, Juntendo University Hospital, Tokyo, Japan. The target number of cases for each clinical trial was set at two, with the total target number of cases for the four studies being eight.

**Table 1:** Outline of four studies

Dental treatment	Tooth extraction	Periodontal regenerative therapy	Maxillary sinus lift	Tooth replantation
Health condition or problem studied	Pericoronitis of wisdom tooth	Chronic periodontitis of moderate severity or greater with vertical periodontal tissue defects	Missing maxillary molars with atrophic maxillary alveolar bone	Fracture of dental root
Target sample size	2	2	2	2
Inclusion criteria	1) Having a wisdom tooth requiring extraction 2) A good systemic condition without chronic or acute diseases 3) Number of platelets >1 × 10^5^/μL 4) Aged ≥ 20 years 5) Other, the investigator believes the patient is suitable for participation in the clinical study	1) Having a periodontal pocket >5 mm at baseline examination 2) Intrabony defect is ≥5 mm in depth, and ≥2 mm in width at the interproximal site of the experimental tooth by X-ray examination 3) Having received initial preparation at the screening 4) Mobility of experimental tooth is 0, 1, or 2, and keratinized gingiva is present 5) Persons whose oral hygiene is established. Good oral hygiene 6) Aged ≥ 20 years 7) Signed informed consent	1) Having a missing tooth in the maxillary posterior region 2) Requiring dental implant treatment at missing tooth site 3) Requiring sinus floor augmentation (socket lift) at the dental implant placement 4) A good systemic condition without chronic or acute diseases 5) Number of platelets >1 × 10^5^/μL 6) Aged ≥ 20 years 7) Other, the investigator believes that the patient is suitable for participation in the clinical study	1) Having a fracture of dental root 2) Having a tooth considered effective by tooth replantation 3) A good systemic condition without chronic or acute diseases 4) Number of platelets >1 × 10^5^/μL 5) Aged ≥ 20 years 6) Other, the investigator believes that the patient is suitable for participation in the clinical study
Exclusion criteria	1) Patients suspected of or having a history of complicated malignant tumors 2) Presence of or a history of abnormal gingival proliferation 3) Patients suspected of oral malignant tumor or precancerous lesion 4) Presence of anticoagulant or anti-platelet medications or bleeding disorders 5) Pregnancy, breastfeeding, or intention of pregnancy 6) Other, the investigator believes that the patient is unsuitable for participation in the clinical study	1) Cannot measure clinical attachment level of experimental tooth 2) History of complicate malignant tumor 3) Suspected oral malignant tumor or precancerous lesion 4) Need to undergo treatment, such as surgical treatment, restorative treatment, or root canal treatment, at the experimental tooth within 36 weeks after transplant 5) Pregnancy, during breastfeeding, or possibility of pregnancy 6) Persons who suffer from disorders of kidney, liver, or blood 7) Disease of the kidneys, liver, and/or blood 8) Active infectious diseases 9) Alcoholism or Drug dependence 10) Mental or consciousness disorder 11) HCV antibody, HBs antigen, ATLA antibody, or HIV antibody positive 12) Smoking more than 10 cigarettes per day 13) Anticoagulant or anti-platelet medications, or a bleeding disorder 14) Other, the investigator believes that the patient is unsuitable for participation in the clinical study	1) Patients suspected of or having a history of complicated malignant tumors 2) Presence of or a history of abnormal gingival proliferation 3) Patients suspected of oral malignant tumor or precancerous lesion 4) Presence of anticoagulant, anti-platelet medications, or bleeding disorders 5) Pregnancy, breastfeeding, or intention of pregnancy 6) Other, the investigator believes that the patient is unsuitable for participation in the clinical study	1) Separated dental root and abscess at the root apical 2) Patients suspected of or having a history of complicated malignant tumors 3) Presence of or a history of abnormal gingival proliferation 4) Presence of anticoagulant or anti-platelet medications or bleeding disorders 5) Pregnancy, breastfeeding, or intention of pregnancy 6) Other, the investigator believes that the patient is unsuitable for participation in the clinical study
Observation period	4 weeks	4 weeks	4 weeks	4 weeks
Follow-up period	As necessary	9 months	6 months	12 months
Primary endpoint	Safety	Safety	Safety	Safety
Secondary endpoints	Clinical evaluation (pain experience) Dental X-ray and/or image evaluation Platelet count	Clinical evaluation (pain experience) Dental X-ray and/or image evaluation Platelet count	Clinical evaluation (pain experience) Dental X-ray and/or image evaluation Platelet count	Clinical evaluation (pain experience) Dental X-ray and/or image evaluation Platelet count

**Table 2:** Common schedule in all four studies

Item	Before registration	Transplantation	After transplantation			
			Observational period			
			1 d	1 wk	2 wk	4 wk
Patient background	●					
Patient agreement	●					
Blood tests for infectious disease	●					
Blood biochemical tests	●					
Hematologic examination	●	●				
Vital sign	●	●				
PRP transplantation		●				
Adverse events		●	●	●	●	●
Concomitant medications		●	●	●	●	●
Interviews for postoperative pain		●	●	●	●	●
Intraoral photography	●	●	●	●	●	●
Dental X-ray	●					●
Sterility testing		●				
Platelet concentration ratio		●				

During the PRP treatment and observational period, observations and tests were conducted 2 weeks before and after the implementation date.

### Inclusion and exclusion criteria

The inclusion criteria for each study are listed in Table 1. Common inclusion criteria were age ≥20 years and meeting the inclusion criteria set by each clinical trial.

The common exclusion criteria were as follows: 1) patients suspected of or having a history of complicated malignant tumors; 2) suspected oral malignant tumor or precancerous lesion; 3) presence of anticoagulant or anti-platelet medications or bleeding disorders; 4) pregnancy, breastfeeding, or intention of pregnancy; and 5) others where the investigator regarding the patient to be unsuitable for participation in the clinical study. The treatment- specific exclusion criteria are shown in Table 1.

### Hematologic examination

Patients satisfying the inclusion criteria were subjected to a hematologic examination as follows: 1) white blood cell (WBC) count, WBC fractions (neutrophils, eosinophils, basophils, and lymphocytes), red blood cell (RBC) count, hematocrit, hemoglobin, and platelet count; 2) blood biochemical tests: aspartate aminotransferase (glutamic- oxaloacetic transaminase), alanine aminotransaminase (glutamic-pyruvic transaminase), total protein, and creatinine; and 3) viral tests: hepatitis C virus (HCV) antibody, hepatitis B surface (HBs) antigen, and human immunodeficiency virus (HIV) antibody.

### PRP preparation

PRP was prepared at the Juntendo University Cell Culture and Processing Facility. After enrollment, on the day of treatment, 20 mL of venous blood was collected using a syringe containing the anticoagulant citrate dextrose solution A (Terumo, Tokyo, Japan). PRP was prepared using a YCELLBIO PRP kit (Ycellbio Medical, Seoul, South Korea) according to the manufacturer’s instructions. All operations involving opening the tube lid were performed on a clean bench (TY-33AD; AS ONE, Osaka, Japan). After collected blood was infused into the YCELL tube, the tube was centrifuged at 1,800 × G for 4 min using a centrifuge (H-19α; KOKUSAN, Saitama, Japan). After two rounds of centrifugation, 1.5 mL of the PRP was carefully withdrawn immediately above the RBC layer. The residual supernatants were collected for sterility testing (SRL; Tokyo, Japan). platelets, RBCs, and WBCs counts in whole blood and PRP were measured using an automated hematology analyzer (XN-1000V; Sysmex, Hyogo, Japan).

Autologous thrombin was prepared at the same time as the PRP. Following blood collection for PRP preparation, 10 mL of venous blood was collected and allowed to stand for 15-20 min. The collected blood was transferred into a 15-mL tube, and centrifuged at 800 × G for 5 min. The supernatant containing autologous thrombin was prepared and added dropwise to the PRP if the gelation was weak. PRP gelation was performed immediately before dental surgery. 0.1 mL 2% calcium chloride (Otsuka Pharmaceutical, Tokushima, Japan) was mixed with approximately 1 mL PRP.

### Health condition or problem studied and dental surgical procedures

Simultaneous to the PRP preparation, the appropriate dental procedure was performed, and the PRP gel was transplanted into the affected area. As a prophylactic measure, the patients were prescribed antibiotics and analgesics after dental surgery. After treatment, the participants were evaluated for safety for 4 weeks. The follow-up periods for each clinical study are shown in Table 1.

#### Post-extraction sockets (socket preservation)

In the post-extraction socket study, the health condition or problem studied was impacted wisdom teeth. Specifically, the participants were patients who had been diagnosed with pericoronitis and had teeth requiring extraction. Thereafter, the post- extraction socket was filled with PRP gel and, as necessary, the flaps were approximated using a surgical suture.

#### Periodontal regenerative therapy

In the periodontal tissue regeneration study, the health condition or problem studied was chronic periodontitis of moderate severity or greater with vertical periodontal tissue loss (intrabony defect with ≥ 5 mm depth and ≥ 2 mm width at the interproximal site). In periodontal flap surgery, after open-flap debridement, the PRP gel was applied to the tooth surface involved in the bone defects, and the flap was approximated using a surgical suture.

#### Maxillary sinus lift

In the maxillary sinus lift study, the health condition or problem studied was missing maxillary molars with atrophic maxillary alveolar bone (missing tooth in the maxillary posterior region requiring dental implant rehabilitation and sinus floor augmentation). After elevation of the flap and access to the sinus was obtained, the maxillary sinus membrane was elevated and PRP gel mixed with synthetic bone graft materials (Cytrans Granules; GC, Tokyo, Japan) was applied. Dental implants were inserted into the PRP transplantation site, and the mucoperiosteal flap was sutured. In Case 1, two dental implants were used (Brånemark System MkIII Groovy R, φ3.75 × 10 mm; Nobel biocare, Zürich-Flughafen, Switzerland). In Case 2, three dental implants were used (Straumann Bone Level Tapered, φ4.1 × 10 mm; Straumann, Basel, Switzerland).

#### Tooth replantation

In the tooth replantation study, the health condition or problem studied was root fracture (fracture of the dental root for which replantation is a viable treatment option and no abscess at the root is present). After tooth extraction, root surface debridement was performed and the dental root was filled with dental materials (Super-Bond, Sun Medical, Shiga, Japan). The PRP gel was administered to the tooth graft site, and the grafted tooth was implanted. The replanted tooth was temporarily fixed to the tooth adjacent to the dental resin (G- FIX, GC).

### Primary endpoint

The primary endpoint of all four studies was safety evaluation. AEs after PRP treatment were evaluated in terms of presence/absence, timing, resolution, extent, treatment, and severity. In addition, AEs were evaluated during the observation period (4 weeks) of PRP transplantation by clinical examination of subjective and objective symptoms and encoded according to the Common Terminology Criteria for Adverse Events (CTCAE version 4.0)^9)^. Furthermore, enrollment in the second case was suspended until the safety evaluation of the first case was complete.

### Secondary endpoints

The common secondary endpoints were 1) clinical evaluation (postoperative pain experience); 2) dental X-ray and/or image evaluation; and 3) platelet count. Postoperative pain was assessed through patient interviews and changes in postoperative pain were evaluated during the observational period (4 weeks). platelet counts in PRP and whole blood (baseline) were measured.

For post-extraction sockets (socket preservation), dental X-rays were taken before surgery and after 4 weeks. For maxillary sinus lift surgery, an additional radiograph was obtained at 6 months after surgery.

For periodontal regenerative therapy, a periodontal examination was conducted, and the clinical attachment level (CAL), probing pocket depth (PPD), tooth mobility, bleeding on probing (BOP), and gingival index were evaluated before surgery and at 4 weeks, 3 months, 6 months, and 9 months after surgery. Dental radiography and cone-beam computed tomography (CBCT) were performed before surgery and at 4 weeks, 3 months, and 9 months after PRP administration. The height of the newly formed alveolar bone at the PRP transplantation site was measured using dental radiography and CBCT.

For tooth replantation, periodontal examinations were conducted; specifically, CAL, PPD, tooth mobility, and BOP were evaluated before surgery and at 3, 6, 9, and 12 months after surgery. Dental radiography was performed before surgery and at 4 weeks, 6 months, and 12 months postoperatively.

### Data collection and analysis

For safety evaluation, the AEs observed in all cases were compiled from each case report form. Data are expressed as mean ± standard division.

## Results

### Enrollment of patients

Two patients were assigned to each of the dental procedures. Eight patients (4 men, 4 women) aged 53.4 ± 14.9 years (range: 27-68 years) were included in the studies.

### Transplanted PRP and quality testing

We measured the PRP and whole blood using an automated hematology analyzer. platelet counts (10^5^/μL, n = 7) were 1.78 ± 0.47 in whole blood and 7.70 ± 3.22 in PRP. The platelet concentration ratio in the PRP was 4.4 ± 1.8. RBC counts (10^4^/μL, n = 7) were 423 ± 71 in whole blood and 4 ± 3 in PRP. The WBC counts (103/μL, n = 7) were 4.6 ± 1.6 in whole blood and 1.5 ± 2.3 in PRP.

The results of visual inspection of foreign matter in the PRP before transplantation and sterility tests were all negative.

### Primary endpoint

During the observation period of 4 weeks after PRP administration, no serious AEs related to PRP therapy occurred. Two AEs were not related to PRP therapy; specifically, one patient presented with diarrhea in the tooth replantation study, and another patient presented with periodontitis outside the PRP transplanted site in the periodontal regenerative therapy study.

### Secondary endpoints

Patient interviews revealed no significant postoperative pain in all four studies. In particular, postoperative pain tended to be suppressed a day after treatment in post-extraction sockets. No postoperative infection at the site of PRP transplantation was observed in any of the eight cases.

**Figure 1:**
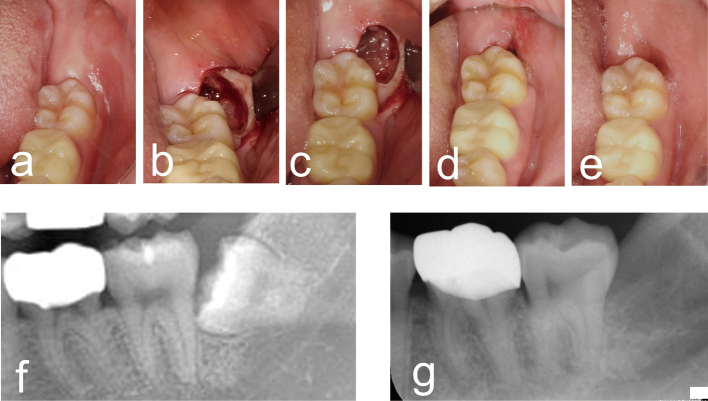
Clinical photographs and panoramic and dental X-ray images of PRP transplantation in a post-extraction socket case A 28-year-old female patient with a lower left wisdom tooth required extraction. Clinical photographs before tooth extraction (a), post-extraction (b), PRP transplantation (c), 1 week (d), and 4 weeks (e) after transplantation. Enlarged panoramic radiograph before tooth extraction (f) and dental radiograph 4 weeks after PRP transplantation (g).

Two patients underwent PRP transplantation into the post-extraction sockets. Good wound healing at the PRP transplant site was observed, without postoperative infection (Figure 1). Wound closure of the post-extraction socket was confirmed 4 weeks after the PRP transplantation (Figure 1e, g).

**Figure 2:**
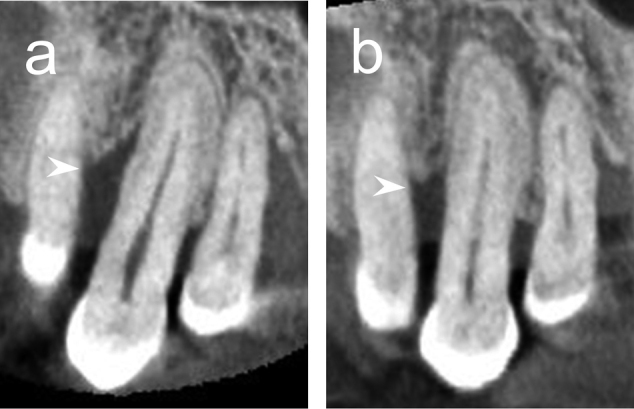
CBCT images before and 9 months after PRP transplantation in a periodontal regeneration therapy case A 66-year-old female patient with chronic periodontitis had 2-3 wall mesial intrabony defects in the right maxillary canine. CBCT images before PRP transplantation (a) and 9 months after PRP transplantation (b). CBCT, cone-beam computed tomography. Arrowheads indicate the PRP transplant site.

Two patients with chronic periodontitis underwent PRP transplantation into the vertical alveolar bone defects. Good wound healing at the PRP transplant site was observed, without postoperative infection (Figure 2). Preoperative depths of alveolar bone defects measured by CBCT were 14.8 mm in Case 1 and 8.6 mm in Case 2, respectively. As a result of PRP transplantation into the vertical alveolar bone defects, the depths thereof were 10.3 mm in Case 1 and 6.7 mm in Case 2.

**Figure 3:**
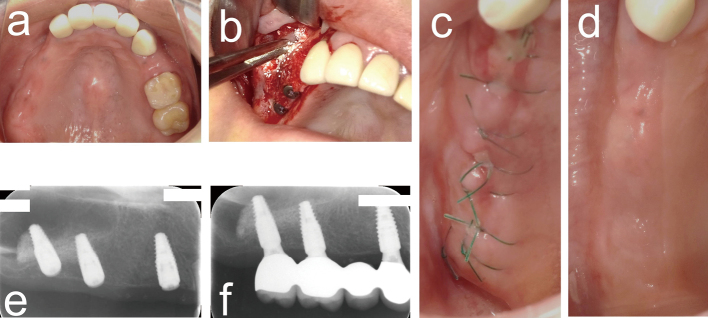
Clinical photographs and dental X-ray images of PRP treatment for a maxillary sinus lift case A 54-year-old female presented with missing right maxillary molars and atrophic maxillary alveolar bone. Clinical photographs before surgery (a), transplantation of a mixture of PRP and synthetic bone graft materials (b), 1 week (c), and 4 weeks (d) after PRP transplantation. Dental radiography images at 6 (e) and 9 months (f) after PRP transplantation.

Following the transplantation of a mixture of PRP and bone graft materials into two patients with missing maxillary molars, good wound healing at the PRP transplant site was observed without postoperative infection (Figure 3). Because the regeneration of hard tissue around the dental implant bodies was confirmed 6 months after PRP transplantation by dental radiography (Figure 3e), the dental implant superstructure was placed 9 months after PRP transplantation (Figure 3f).

**Figure 4:**
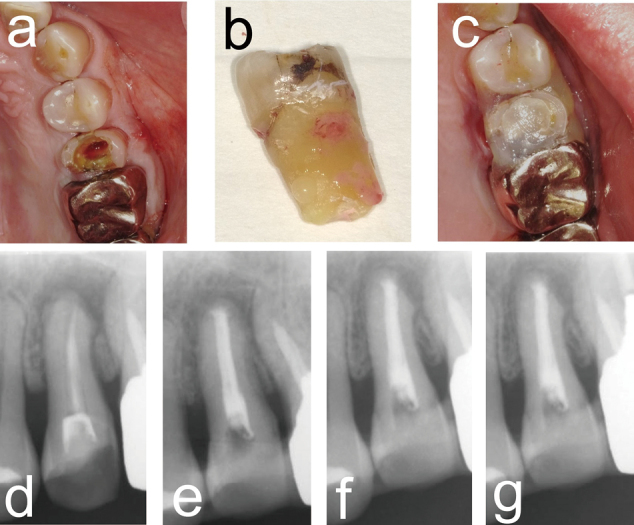
Clinical photographs and dental X-ray images of PRP treatment for a tooth replantation case A 49-year-old male presented with a dental root fracture of the upper left premolar. Clinical photographs before surgery (a), the extracted tooth (b), and immediately after PRP transplantation and replantation of the restored tooth (c). Dental radiographs before surgery (d) and at 3 (e), 6 (f), and 12 months (g) after PRP transplantation.

In two patients with root fractures, the fractured tooth was extracted and debridement of the socket was performed. The PRP gel was transplanted into the socket and the restored tooth was replanted and fixed to the adjacent tooth (Figure 4c). Following PRP transplantation, there was no postoperative infection and wound healing at the PRP transplant site was good (Figure 4). Twelve months after PRP transplantation, hard tissue regeneration was confirmed around the root of the replanted tooth (Figure 4g).

## Discussion

PRP therapy is widely performed in dentistry^4, 5)^, and numerous studies showed the validity of PRP^5)^. In dentistry, PRP-related products such as platelet-rich fibrin (PRF)^10)^, and plasma rich in growth factor (PRGF)^11, 12)^, and concentrated growth factor (CGF)^13)^ are also used. Regarding the safety of PRP transplantation for the four dental diseases, no AEs were observed in any case. Although the sample size was limited, we confirmed the safety of the PRP prepared in this study for each dental disease.

PRP is widely used for pain relief and wound healing. Although PRP therapy is a simple procedure, various AEs suspected to be related to PRP treatment, including postoperative infections, have been reported in recent years^7)^. To date, the causal relationship between each AEs and PRP treatment has not been fully elucidated. Therefore, it is important to assess the safety of PRP transplantation for each target disease.

Regarding the validity of PRP for each dental disease, postoperative pain tended to be mild in all cases. In particular, postoperative pain in the post-extraction sockets tended to subside from the day after treatment. Additionally, the incidence of alveolar osteitis, also known as dry socket, is more frequent with mandibular third-molar extraction^14)^. Transplantation of PRP into post-extraction sockets significantly reduces incidence of alveolar osteitis compared to controls^15-17)^. In our study, no incidence of alveolar osteitis was observed after tooth extraction, suggesting that PRP transplantation may have had a preventive effect against the onset of alveolar osteitis after tooth extraction. From the perspective of preventing the onset of alveolar osteitis and subsiding postoperative pain, transplantation of PRP into the post-extraction sockets may be effective.

Hard tissue regeneration at the PRP transplantation site was observed during periodontal tissue regeneration, maxillary sinus lift, and tooth replantation. Furthermore, the stability of the tooth or dental implant body was observed 9 months after PRP transplantation. Taken together, PRP transplantation may promote wound healing and be useful to treat dental diseases.

There are several clinical challenges remaining regarding the popularization of PRP therapy. First, the complexity of the procedure on the day of surgery is a challenge, as blood collection and preparation of PRP are performed at the same time. Second, as PRP is administered immediately after preparation, it is impossible to confirm its sterility prior to administration. Postoperative infection is one of the most frequently reported AEs related to PRP therapy^7)^, presumably due to inappropriate hygiene control during PRP treatment^18^^-^^20)^. Therefore, it is important to implement rapid microbial methods even for PRP therapy. However, since PRP can be processed and administered to patients within a few hours, quality testing also needs to be completed in a short time. In the future, it will be necessary to develop rapid microbial methods suitable for clinical use of PRP.

To overcome these issues, it may be clinically useful to manufacture and freeze PRP in advance and then thaw it on the day of the transplant. The cryopreservation of PRP can reduce the workload on the day of surgery and allow time for sterility testing, ensuring greater safety. Recently, several groups have investigated the effects of cryopreservation on the properties of PRP^21-25)^. Several groups have shown that some cytokine levels in cryopreserved PRP are higher than those in fresh PRP^22, 23, 25)^. Additionally, studies have been conducted on the administration of cryopreserved PRP, and the results support the possibility that cryopreserved PRP administration may also be effective^26-28)^. The cryopreservation of PRP may be clinically relevant in terms of its efficacy.

In this study, we assessed the safety and validity of PRP therapy in dentistry. Despite the small sample size, we confirmed the safety of periodontal surgery with PRP transplantation for each type of dental disease. Additionally, the administration of PRP into the extraction socket contributed to pain relief the day after tooth extraction, which was considered to have a degree of validity. Further large-scale clinical studies are required to evaluate the safety and efficacy of PRP for each type of dental disease.

## Funding

This research received no external funding.

## Author contributions

Conceptualization: MT; methodology: YM; software: YM; validation: KW; formal analysis: MT; PRP preparation: MT, YM, AA; investigation: MT, SN, MH, TT, and MS; resources: MT; data curation: KW, HY; writing-original draft preparation: MT, AA; writing-review and editing: MT, AA; visualization: YM; supervision: MT; project administration: YM and KW. All the authors have read and approved the final version of the manuscript.

## Conflicts of interest statement

The authors declare that there are no conflicts of interest.
